# Case report: A case of adolescent invasive encapsulated follicular variant of papillary thyroid carcinoma with literature review

**DOI:** 10.3389/fonc.2026.1780439

**Published:** 2026-06-30

**Authors:** Wenxuan Zhang, Nina Qu

**Affiliations:** 1 Medical Imaging Institute, Shandong Second Medical University, Weifang, China; 2 Ultrasound Department, Yantai Yuhuangding Hospital, Yantai, China

**Keywords:** invasive encapsulated follicular variant of papillary, thyroid cancer, adolescent, ultrasound, case report

## Abstract

Invasive Encapsulated Follicular Variant of Papillary Thyroid Carcinoma (IEFVPTC) is a well-differentiated malignant thyroid tumor. The 2022 WHO 5th edition classification of endocrine tumors recognizes it as an independent entity. It is more common in adults and rare in children. This report describes a 13-year-old female patient who presented with a neck mass. Ultrasound revealed a 3.4 cm×2.2 cm×2.5 cm hypoechoic nodule in the left lobe, exhibiting regular shape, well-defined margins, an aspect ratio <1, heterogeneous internal echoes, and hypervascularity, corresponding to TI-RADS 4a. Radionuclide imaging demonstrated a “hot nodule”. Following preoperative oral methimazole therapy to control thyroid function, the patient underwent “total endoscopic transaxillary left thyroid lobectomy”. Pathology confirmed IEFVPTC (minimally invasive type). A review of the literature shows that IEFVPTC may present with benign ultrasound features, leading to misdiagnosis. Heightened vigilance is warranted for hypervascular nodules in adolescents, with needle biopsy indicated when necessary. Lobectomy suffices for tumors <4 cm without vascular invasion.

## Introduction

A 13-year-old female patient was admitted to our hospital’s thyroid surgery department, presenting with a “pre-sternal neck mass discovered over one month ago”.

**Figure 1:**
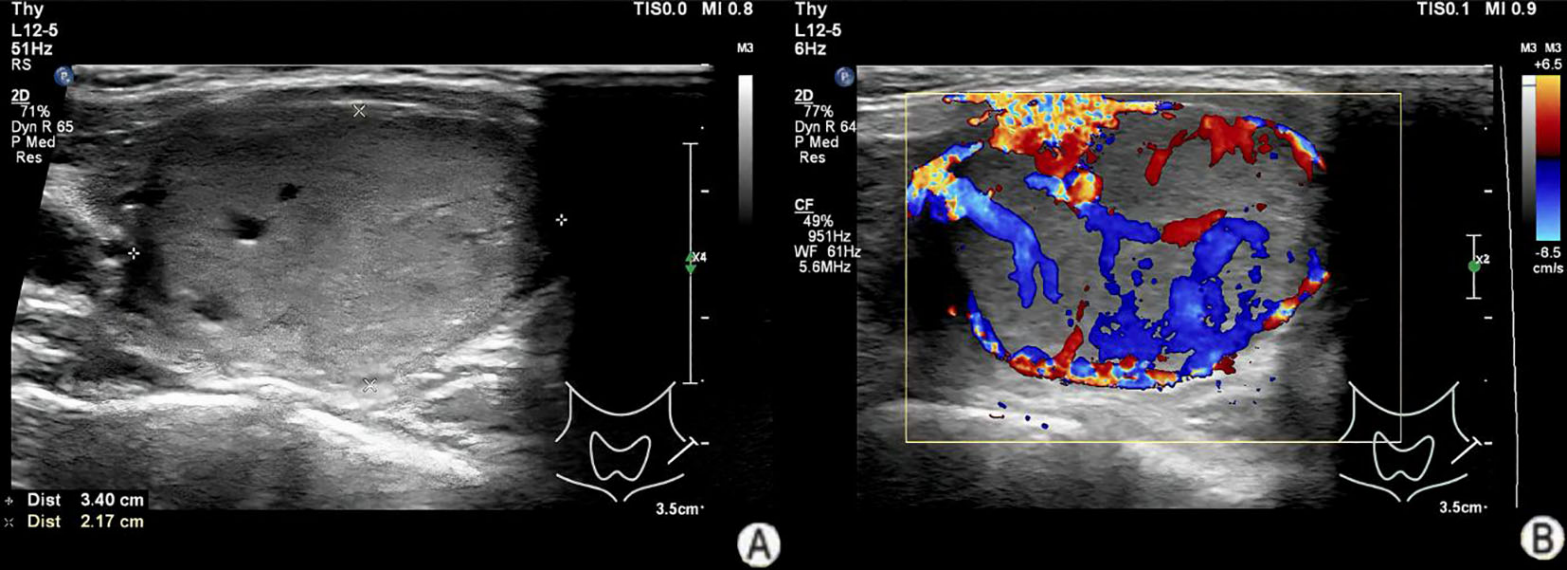
Thyroid ultrasound image: A hypoechoic nodule measuring approximately 3.4 cm × 2.2 cm × 2.5 cm is visible in the middle-lower portion of the left thyroid lobe. It exhibits a regular shape, clear margins, a length-to-width ratio <1, and heterogeneous echogenicity **(A)**. Color Doppler flow imaging (CDFI) demonstrates abundant internal blood flow signals **(B)**.

**Figure 2:**
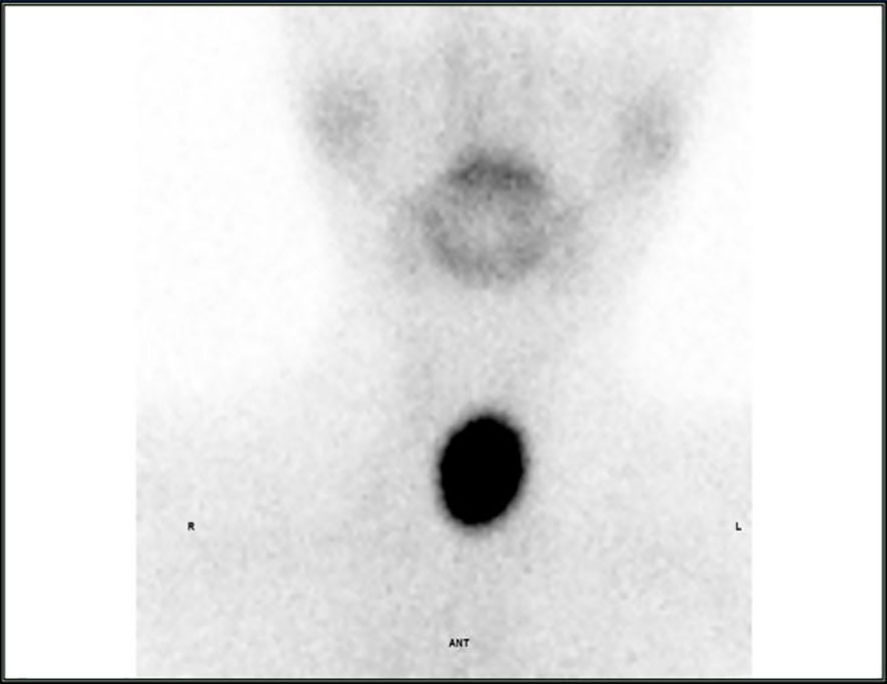
Thyroid radionuclide scan: “Hot nodule” in the left thyroid lobe.

The patient was initially admitted, presenting with “a pre-sternal mass for 2 weeks and sore throat for 5 days”. Physical examination: Height: 168 cm, Weight: 58 kg, BMI: 20.55 kg/m², Finger-to-floor reach: 169 cm, Seated height: 87 cm, slight proptosis, grade II enlargement of the left thyroid lobe with a palpable 2 cm × 3 cm nodule without tenderness, no lymph node enlargement was detected, no abnormalities in the right thyroid lobe, slender fingers, and no tremor with arms extended horizontally. Routine ECG revealed sinus tachycardia. Laboratory thyroid panel results: Thyroid stimulating hormone 0.016 mIU/L (reference range: 0.51-4.17 mIU/L), Free thyroxine 24.00 pmol/L (reference range: 12.60-21.00 pmol/L), Free triiodothyronine 9.23 pmol/L (reference range: 3.94-7.67 pmol/L), Anti-thyroglobulin antibody 152.1 IU/L (reference range: 0–115 IU/L), Anti-thyroid peroxidase antibody 20.89 IU/mL (reference range: 0–34 IU/mL), Thyroid-stimulating hormone receptor antibody 1.33 U/L (reference range: 0-1.75 U/L). Ultrasound examination: A nodule measuring approximately 3.4 cm × 2.2 cm × 2.5 cm was detected in the middle-lower portion of the left thyroid lobe. It exhibited a regular shape with clear margins, an aspect ratio < 1, and heterogeneous internal echoes. No significant posterior acoustic enhancement was observed. Rich blood flow signals are visible within the nodule (**Figure 1**), no lymph node enlargement was detected. The nodule was classified as TI-RADS 4a. Thyroid scintigraphy: The “hot nodule” in the left thyroid lobe showed increased 24-hour iodine uptake (**Figure 2**). The patient was instructed to take “methimazole, 10mg qd” orally to control thyroid function. Surgery was performed after thyroid function was well controlled.

**Figure 3:**
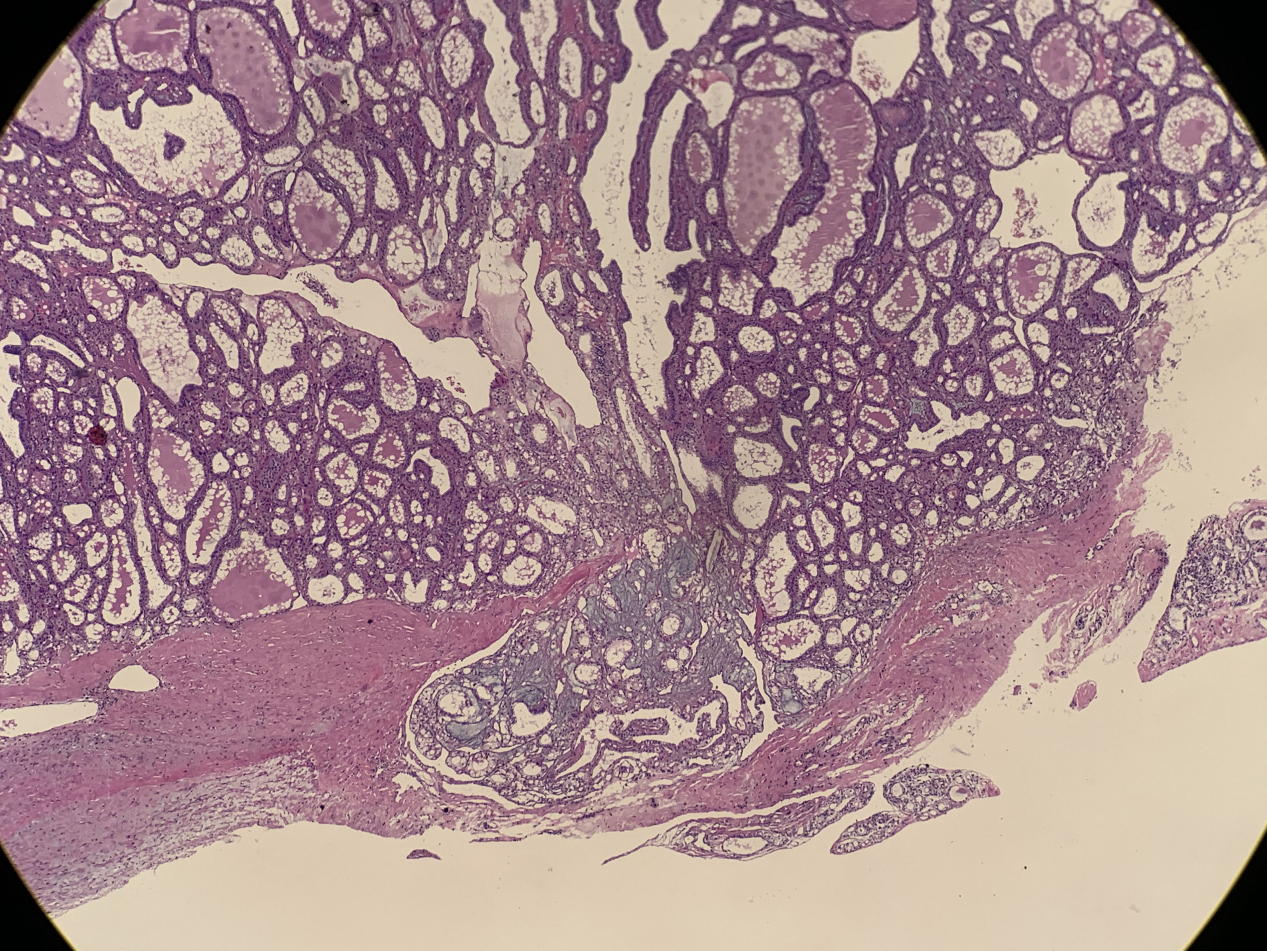
Pathology image: Thyroid follicular tumor, Partial cytoplasmic eosinophilia. Some follicular epithelial cells exhibit RAS-like nuclear features. Capsular invasion is visible; no significant vascular invasion is noted.

The patient was readmitted a month later. Follow-up thyroid function tests showed well-controlled levels: Thyroid stimulating hormone 0.705 mIU/L, Free thyroxine 8.65 pmol/L, Free triiodothyronine 3.50 pmol/L. The patient underwent total endoscopic transaxillary left thyroid lobectomy with intraoperative parathyroid identification. Intraoperative laparoscopic findings: A mass approximately 3.5 cm × 2.5 cm was palpated in the midportion of the left thyroid lobe. It was firm in consistency with clear borders and an intact capsule. No invasion of the thyroid capsule was noted, and no enlarged lymph nodes were identified. Pathological Diagnosis: left thyroid follicular tumor. Partial cytoplasmic eosinophilia. Some follicular epithelial cells exhibited RAS-like nuclear features. Capsular invasion was visible. No significant vascular invasion was noted (**Figure 3**). The findings were consistent with Invasive Encapsulated Follicular Variant of Papillary Thyroid Carcinoma (minimally invasive type). The patient has had thyroid function tests every three months since the surgery; so far, all results have been normal, and there have been no complications.

Discussion: Invasive Encapsulated Follicular Variant of Papillary Thyroid Carcinoma (IEFVPTC) is a well-differentiated malignant thyroid tumor. In the 5th edition of the WHO Classification of Endocrine Tumors published in 2022, IEFVPTC is no longer classified as a subtype of papillary thyroid carcinoma (PTC) but is recognized as an independent entity, and is listed it as a separate malignant tumor of follicular cell origin (1, 2). Its core significance lies in the fundamental difference between its RAS-like molecular profile and the BRAF-like features of classic PTC, with clinical behavior resembling follicular carcinoma—predominantly hematogenous metastasis, rare lymph node involvement, and relatively indolent biology. As an independent entity, its diagnostic criteria now incorporate an invasiveness grading system akin to follicular carcinoma, enabling precise risk stratification and preventing both overtreatment of low-risk lesions and undertreatment of malignant ones. Clinically, surgical extent should be tailored to invasiveness degree, with greater emphasis on distant metastasis screening rather than lymph node status alone, given its hematogenous spread tendency (3, 4).

IEFVPTC predominantly affects adults, with a median age at diagnosis between 40 and 50 years. Pediatric cases are extremely rare, and the female-to-male patient ratio exceeds 3:1 (5). This case involves a 13-year-old adolescent female, representing a rare report.

Ultrasound examination is the preferred screening and diagnostic modality for thyroid tumors. IEFVPTC exhibits features characteristic of both benign and malignant nodules on ultrasound imaging. It typically appears as a round or oval lesion with well-defined, smooth margins. A pseudocapsule may be visible in some cases. Internally, it presents as isoechoic or hypoechoic, usually without microcalcifications. The aspect ratio is typically < 1. Peripheral blood flow predominates, with minimal internal blood flow (6, 7). The conventional ultrasound findings in this case align with the characteristics mentioned earlier, but abundant internal blood flow signals were observed. Conventional ultrasound lacks specificity; definitive diagnosis still requires fine-needle aspiration biopsy or histopathological examination, along with molecular testing.

The pathological diagnostic criteria for IEFVPTC represent a key update in the 5th edition of the WHO Classification of Endocrine Tumors. Its histological features include a complete capsule, predominant follicular architecture, papillary nuclear features, and potential capsular or vascular invasion. The core diagnostic elements are “follicular growth pattern and papillary nuclear features, intact capsule with vascular or capsular invasion” (2).

The National Comprehensive Cancer Network (NCCN) 2024 clinical practice guidelines classify IEFVPTC as a subtype of PTC, with treatment fully stratified based on tumor size, invasion, and the number of lymph node and vascular infiltrates: For tumors ≤4cm and confined to the gland, lobectomy and isthmus resection are recommended; Tumors >4cm, macroscopically invasive tumors, clinically positive lymph nodes, or intraoperative findings of extensive vascular invasion (≥4 vessels or macroscopic thrombus) require total thyroidectomy, central zone dissection of the affected region and I-131 with strict TSH suppression (8). In this case, the tumor was <4cm, confined to the middle-lower portion of the left thyroid lobe, and showed no vascular invasion. Left thyroid lobectomy was performed according to the guidelines.

Although conventional ultrasound revealed low-risk features—regular shape, clear margins, and a longitudinal-to-transverse ratio < 1, this case exhibited rich internal blood flow and heterogeneous echogenicity. Additionally, the patient was a 13-year-old adolescent female. Therefore, for such atypical, hypervascular thyroid nodules, even with a strong benign tendency, vigilance for the possibility of invasive encapsulated follicular subtype papillary carcinoma is warranted. Ultrasound-guided biopsy should be performed to guide the precise clinical diagnosis and treatment for low-risk groups such as adolescents.

According to published literature, the incidence of PTC in children is 0.46 cases per 100,000 population (9), with approximately 15% of these cases belonging to the IEFVPTC subtype, indicating that this subtype is indeed relatively rare in children (10). To date, no clear association has been identified between IEFVPTC and hyperthyroidism or positive thyroid antibodies, nor have any articles describing the ultrasound findings of IEFVPTC been found.

## Data Availability

The original contributions presented in the study are included in the article/supplementary material. Further inquiries can be directed to the corresponding author.

## References

[1] Basolo F Macerola E Poma AM Torregrossa L . The 5th edition of WHO classification of tumors of endocrine organs: changes in the diagnosis of follicular-derived thyroid carcinoma. Endocrine. (2023) 80:470–6. doi: 10.1007/s12020-023-03336-4 36964880 PMC10199828

[2] Baloch ZW Asa SL Barletta JA Ghossein RA Juhlin CC Jung CK . Overview of the 2022 WHO classification of thyroid neoplasms. Endocr Pathol. (2022) 33:27–63. doi: 10.1007/s12022-022-09707-3 35288841

[3] Cancer Genome Atlas Research Network . Integrated genomic characterization of papillary thyroid carcinoma. Cell. (2014) 159:676–90. doi: 10.1016/j.cell.2014.09.050 PMC4243044 25417114

[4] Nikiforov YE Seethala RR Tallini G Baloch ZW Basolo F Thompson LD . Nomenclature revision for encapsulated follicular variant of papillary thyroid carcinoma: a paradigm shift to reduce overtreatment of indolent tumors. JAMA Oncol. (2016) 2:1023–9. doi: 10.1001/jamaoncol.2016.0386 27078145 PMC5539411

[5] Jin S Xie L Zhang G Liu L Xia K Liu H . Prognosis of invasive encapsulated follicular variant and classical papillary thyroid carcinoma: a propensity score-matched study using the SEER database. Sci Rep. (2025) 15:413. doi: 10.1038/s41598-024-84425-w 39747560 PMC11696095

[6] Rui C . Ultrasound imaging and clinical characteristics of thyroid follicular papillary carcinoma. Yingxiang Yanjiu yu Yixue Yingyong. (2022) 6:73–5. Rui C. Ultrasound imaging and clinical characteristics of thyroid follicular papillary carcinoma [J]. Yingxiang Yanjiu yu Yixue Yingyong, 2022, 6(21): 73-75. (in Chinese).

[7] Wu Y Qi X Zhou A Qi Q Li Y Xu P . Sonographic and clinical features of encapsulated versus infiltrative follicular variant papillary thyroid carcinoma. Zhongguo Chaosheng Yixue Zazhi. (2023) 39:966–9. Wu Y, Qi X, Zhou A, et al. Sonographic and clinical features of encapsulated versus infiltrative follicular variant papillary thyroid carcinoma [J]. Zhongguo Chaosheng Yixue Zazhi, 2023, 39(09): 966-969. (in Chinese).

[8] Haddad RI Bischoff L Ball D Bernet V Blomain E Busaidy NL . Thyroid Carcinoma, Version 2.2022, NCCN Clinical Practice Guidelines in Oncology. J Natl Compr Canc Netw. (2022) 20(8):925–51. doi: 10.6004/jnccn.2022.0040 35948029

[9] Moleti M Aversa T Crisafulli S Trifirò G Corica D Pepe G . Global incidence and prevalence of differentiated thyroid cancer in childhood: systematic review and meta-analysis. Front Endocrinol (Lausanne). (2023) 14:1270518. doi: 10.3389/fendo.2023.1270518 37795368 PMC10546309

[10] Jin S Xie L Zhang G . Prognosis of invasive encapsulated follicular variant and classical papillary thyroid carcinoma: a propensity score-matched study using the SEER database. Sci Rep. (2025) 15(1):413. doi: 10.1038/s41598-024-84425-wIF 39747560 PMC11696095

